# The Role and Mechanisms of Traditional Chinese Medicine for Airway Inflammation and Remodeling in Asthma: Overview and Progress

**DOI:** 10.3389/fphar.2022.917256

**Published:** 2022-07-15

**Authors:** Bo-wen Zhou, Hua-man Liu, Xin-hua Jia

**Affiliations:** ^1^ Shandong University of Traditional Chinese Medicine, Jinan, China; ^2^ Affiliated Hospital of Shandong University of Traditional Chinese Medicine, Jinan, China

**Keywords:** asthma, TCM, airway inflammation, airway remodeling, autophagy

## Abstract

Asthma as an individual disease has blighted human health for thousands of years and is still a vital global health challenge at present. Though getting much progress in the utilization of antibiotics, mucolytics, and especially the combination of inhaled corticosteroids (ICS) and long-acting β-agonists (LABA), we are confused about the management of asthmatic airway inflammation and remodeling, which directly threatens the quality of life for chronic patients. The blind addition of ICS will not benefit the remission of cough, wheeze, or sputum, but to increase the risk of side effects. Thus, it is necessary to explore an effective therapy to modulate asthmatic inflammation and airway remodeling. Traditional Chinese Medicine (TCM) has justified its anti-asthma effect in clinical practice but its underlying mechanism and specific role in asthma are still unknown. Some animal studies demonstrated that the classic formula, direct exacts, and natural compounds isolated from TCM could significantly alleviate airway structural alterations and exhibit the anti-inflammatory effects. By investigating these findings and data, we will discuss the possible pathomechanism underlined airway inflammation and remodeling in asthma and the unique role of TCM in the treatment of asthma through regulating different signaling pathways.

## Introduction

Bronchial asthma, characterized by bronchoconstriction, is a common chronic respiratory disease that negatively influences the quality of life of patients worldwide and the population of patients doubles every 10 years without any definite reasons, especially in developing countries (Suk et al., 2016). In asthma, previous research focused more on big airways because of their obvious changes and easy access for biopsy. However, increasing evidence shows that small airway (<2 mm in diameter) dysfunction, which involves airway inflammation and remodeling, is a key relevant factor in the pathogenesis of asthma, which also represents a trademark of asthma persistence. Based on a large prospective study (Postma et al., 2019), the chronic soakage of inflammatory cells and its pathway was involved in almost all asthma severities and aggravated the abnormalities in airway function and structure. Compared with healthy lungs, mucus-secreting goblet cells in the epithelium have been frequently observed in the small airways of patients with severe asthma according to the flatter of inflammatory mediators (van den Bosch et al., 2021). Such structural changes are collectively termed “airway remodeling.” The cause(s) of airway remodeling is still unknown though airway inflammation has long been considered the culprit. It is suggested that airway smooth muscle epigenetic changes (Kaczmarek et al., 2019), autophagy (Theofani and Xanthou, 2021), and immune cells (Guida and Riccio, 2019) are drivers of remodeling, which indicates that both airway inflammation and remodeling are independent factors of asthma fatal attacks.

Currently, asthma endotypes have been defined and broadly regarded as type 2 (T2) high or T2 low based on the classical description of immediate or late allergic airway disorders. These two different types correspond to either eosinophilic asthma or non-eosinophilic phenotypes (Carr et al., 2018). The combined administration of β2 adrenoceptor agonists and inhaled corticosteroids, as well as biological therapy (Costello and Cushen, 2020), has been widely applied in clinical circumstances for chronic airway inflammation and remodeling. However, not only these therapies above may cause severe side effects but also quite a few patients without the rise of eosinophilic granulocyte in blood have been observed to have airway inflammation and remodeling in the biopsy. Therefore, it is necessary to explore more therapies for asthma, aiming at alleviating continuous airway inflammation and remodeling. Traditional Chinese Medicine (TCM) has been applied to the clinical treatment of asthma for thousands of years in China. Intriguingly, recent reports have found that multiple bioactive components isolated from Chinese herbal and formulas based on TCM theories could exhibit anti-inflammatory and anti-remodeling effects, such as Formononeti (Yi et al., 2020). Such findings suggest a potential of TCM in airway inflammation and remodeling therapy via modulating the immune system or autophagy. In this review, we will briefly discuss the driver mechanisms underlying airway inflammation and remodeling in asthma. The TCM theories for treating the disease and moderating symptoms will be described. And, we also discuss the formulas, extracts, and active ingredients with potential in anti-inflammation, anti-remodeling, and their roles in asthma. Further discussion on TCM will focus on the outlooks for future research and the challenges of utilizing TCM to moderate airway symptoms of asthma, which intend to enlighten the new therapies or strategies on small airway dysfunction.

### Airway Inflammation and Remodeling Factors

Asthma is a chronic heterogeneous disease that is usually characterized by airway inflammation, obstruction, and airway hyperresponsiveness (AHR). Research (Wang C. et al., 2018) demonstrated that the increased airway smooth muscle (ASM) thickness and allergy contribute independently and additively to AHR, which is the primary structural abnormality of asthma. Meanwhile, the changes in ASM are one of the most crucial pathological progresses in airway remodeling. Although it has been difficult to confirm, it (Grainge and Park, 2018) has been seen in some asthmatics that airway remodeling contributes to the development of fixed airway obstruction. The aggravation of airway inflammation and remodeling is associated with the rapid attack of fatal asthma but the mechanisms and driver factors of them are still unknown.

The pathogenesis of inflammation in asthma is influenced by multiple factors, with environmental factors such as smoking, temperature, chemical irritants, and body weight abnormalities being the key elements leading to asthma progression (Liu et al., 2021). These factors above may boost the release of cytokines, allergens, chemokines, and infectious agents, which will activate signaling pathways in epithelial cells in asthma (Aghasafari et al., 2019). The majority of asthma patients show atopic symptoms and have allergic airway inflammation extending from the trachea down to peripheral airways. In general, Toll-like receptors (TLRs), existing on the membrane surface (Tripathi and Aggarwal, 2006), recognize associated molecular patterns and activate inflammatory cells like nuclear factor kappa-light-chain-enhancer of activated B-cells (NF-κB). Then, the resolution process starts where antigen-presenting cells (APCs) endocytose inhaled allergens, which indirectly participate in activating mast cells by crosslinking surface-bound immunoglobulin E (IgE) molecules to release several bronchoconstrictor mediators. Moreover, many known immune signaling pathways, such as mitogen-activated protein kinase (MAPK) and nuclear factor erythroid-2-related factor 2/heme oxygenase-1(Nrf2/HO-1) are specifically associated with airway inflammation in response to various factors (Wang K. C. W. et al., 2018). Peter J. Barnes (2017) found that the increased release of mediators from inflammatory cells (particularly mast cells) may induce the attack of AHR in asthma. According to the existing research (Wang J. et al., 2021), the multiple active ingredients from Chinese medicinal plants are potential treatment strategies in controlling AHR by reducing Th2 cytokines, modulating Th1/Th2, and suppressing inflammatory pathways, which systemically confirmed the effectiveness of TCM on airway anti-inflammation.

Airway remodeling is characterized by structural alternatives, consisting of increased mucus-secreting goblet cells in the epithelium, thickening of the sub-epithelial collagen layer, angiogenesis, as well as the increase of ASM mass and volume (Grainge and Park, 2018). Within the classical description of the relationship between airway inflammation and remodeling, some of the known Th2 highly induced factors, such as eosinophils, neutrophils, cytokines, chemokines, mast cells, and growth factors, contribute to the thickness of the epithelium, the basement membrane (BM), the subepithelial layer, and ASM (Bergeron and Boulet, 2006). These structural changes have been found in both big and small airways, yet not easy to demonstrate. Further, in a recent review, Guida (Guida and Riccio, 2019) pointed out a complex rather than single relationship between airways remodeling and inflammation, which are not limited to Th2-induced inflammatory effects but also induced, suppressed, or regulated by different cellular and molecular pathways, especially the extracellular matrix (ECM). ECM plays a unique role in the structural stability of the airways wall being consisting of a network of collagenous and non-collagenous ECM protein surrounding cells in the airways. Meanwhile, the lung ECM remodeling starts from the ongoing deposition of proteins, basically because of the transforming growth factor-beta (TGF-β) signaling. Confirmed by *in vivo* and *in vitro* trials (Saito et al., 2018), TGF-β increases ASM proliferation by activated fibroblasts and has been promoted by uncontrolled bronchoconstriction in asthma. Other factors such as matrix metalloprotein (MMP), especially MMP-9, MMP-12, or Rho-kinase (ROCK) proteins, contribute to the remodeling process as well by degrading the components of the ECM. Several studies (Bortolozzo et al., 2018; Righetti et al., 2014) using ovalbumin (OVA)-induced models of asthma have shown functional and structural alterations in the respiratory system associated with the high expression of TGF-β, MMP-9, MMP-12 positive cells, and Th2 cytokines: the increased deposition of actin and elastic fibers, and increased mucus production among big and small airways. The functional consequences of these alterations mostly result in the obstruction of small airways, airways narrowing, and the depressing of lung function due to sputum secretion (Ito et al., 2019). However, despite the widespread utilization of bronchodilators and corticosteroids to relieve the symptoms of asthma airways remodeling, there are no clinical medicines that have shown a significant efficacy to reverse all changes mentioned above. Fortunately, an increasing number of trials suggested the effective treatment of TCM therapies for airway anti-remodeling. Wieczfinska et al. (2020) have justified that the *Leonurus sibiricus* root extracts could significantly decrease airways remodeling marker expression via *in vivo* studies.

**FIGURE 1:**
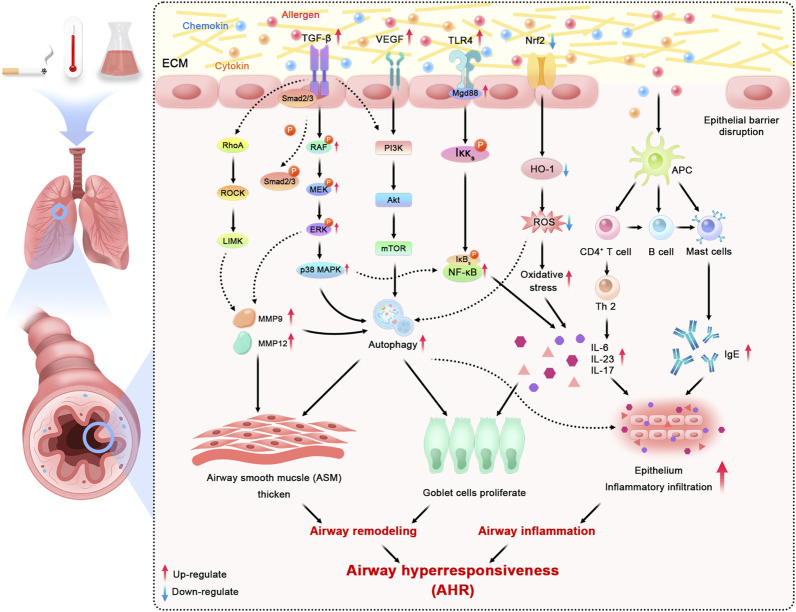
Schematic overview of major mechanisms and related signaling pathways in asthmatic inflammation and airway remodeling (ECM: extracellular matrix; TGF-β: transforming growth factor-beta; VEGF: vascular endothelial growth factor; TLR4: Toll-like receptor 4; Nrf2: nuclear factor erythroid-2 related factor 2; RhoA: ras homolog family member A; ROCK: rho-associated coiled-coil containing protein kinase; LIMK: LIM domain kinase; MAPK: mitogen-activated protein kinase; PI3K: phosphatidylinositol-4,5-bisphosphate 3-kinase; Akt: AKT serine/threonine kinase; mTOR: mechanistic target of rapamycin kinase; NF-κB: nuclear factor kappa B; HO-1: hemeoxygenase-1; ROS: reactive oxygen species; APC: antigen presenting cell; MMP: matrix metallopeptidase; IL: interleukin; IgE: immunoglobulin E).

Among recent studies, the unbalance of autophagy has been widely demonstrated in allergic airway inflammation. As a vital regulator of fibrosis, autophagy can strongly enhance ECM production in ASM and mesenchymal cells, contributing to airway thickening and rigidity. Consistently, based on previous studies (Joean et al., 2017; Choi et al., 2018), the autophagy inhibition in pulmonary inflammation may help to preserve the maintenance of lung homeostasis and the control of fatal inflammatory responses by decreasing the exposure of immune factors, such as IL-17, IL-23, and TLR4. Similarly, the epithelial cells depleted autophagy-related 5 or autophagy-related 4 genes exerted a block in mucus generation with IL-13, which indicated that autophagy is critical for airway mucus generation and Th2 response in asthma (Dickinson et al., 2016). McAlinden et al. 2019) have shown that inhabitation autophagy reduces airways remodeling and alleviates bronchoconstriction in a TGFβ1-dependant manner through building murine models. The potential pathological mechanism of airway alterations is also associated with the close relationship between abnormal autophagy and dysregulation of redox homeostasis (Ornatowski et al., 2020). The activation of oxidative stress results in increases in epithelial vascular permeability and ASM contraction (Kleniewska and Pawliczak, 2017). Overall, blocking and moderating autophagy have been regarded as an attractive target to moderate airway remodeling and inflammation in asthma. All major mechanisms mentioned above in asthmatic airway inflammation and remodeling can be consulted in Figure 1.

As one of the main choices of complementary and alternative therapy for asthma, TCM has extensive prospects in reversing autophagy to control asthma serve attacks. The treatment of Shensuyin (Zhang H. et al., 2021), a traditional herbal formula, has been confirmed by its inhibition in the PI3K/Akt/mTOR signaling pathway, which is consistent with the induction of autophagy. A large number of single herbals from TCM show their potential possibilities as autophagy modulators providing a direct link from traditional therapies to modern treatment in asthma.

### Theoretical Research in Inflammation and Remodeling of TCM

TCM, consisting of therapies and methods, has been widely applied in treating asthma in East and South Asia for a few thousand years as an effective medical system (Ou Yang and Gu, 1996). Unlike evidence-induced modern medicine (Chan and Ng, 2020), TCM is based on the clinical experience about the etiology of diseases, the diagnosis of disease syndromes, prevention, and treatments. In ancient theory, asthma was called wheezing disease for its pulmonary symptoms like cough, sputum, and wheezing sound. According to theoretical studies of TCM, it has been found that maintaining the lung function requires a relative balance between a smooth flow of energy (commonly called “Qi”) and blood. Only the smooth circulation of Qi and blood can ensure the respiratory function of the lung. Chinese Herbal medicine (CHM) believes that asthma is associated with the Qi deficiency in the lung, which directly leads to the disturbance of blood flow. The main courses of Qi deficiency could be attributed to congenital weakness or rapid chronic attack of illness. Meanwhile, when the lung is deficient, not blood but also other body fluids will hamper the body and become sputum blockage and blood stasis based on the TCM. It (Liu et al., 2021) is confident to be confirmed that these alterations are similar to chronic inflammatory progress and angiogenesis in airways remodeling.

In TCM theory, it was found that there is a close relationship between lungs and kidneys’ physiology. Once the kidney is deficient, the lung function will also be restrained. Thus, the major treatment therapeutic method of TCM aims to invigorate kidney function and strengthen the flow of Qi for asthma patients to relieve symptoms (Geng et al., 2016). Further, the TCM summarizes relative symptoms of airways inflammation as well as remodeling in asthma and has classified it as a stagnation of qi and blood stasis syndrome or phlegm-heat pattern. Thus, the aim of management of asthma is formulated by TCM therapy principles of removing blood stasis (quyu), benefiting the Qi (yiqi), clearing the heat (qingre), and eliminating sputum (qutan).

At present, the obvious advantages of TCM in asthma treatment and control have been given increasing attention and clinical proof. Several observational studies (Wang et al., 2014) in China on more than 360 patients suggested that the addition of TCM to conventional therapy alleviated acute symptoms and reduced drug use. One meta-analysis (Song et al., 2016) over 20 RCTs involving 1,590 participants evaluated the safety and efficacy of CHM in treating childhood cough variant asthma (CVA) and demonstrated that there could be an additive benefit from CHM in terms of improving the induces among CVA patients. Normally, CVA patients present intractable cough and lung function alteration (mainly the forced expiratory volume in 1 second, FEV_1_) along with the increase of LgC5 and IgE. The study has shown that the CHM group had a positive effect in improving FEV_1_ and LgC5 as well as the falling total IgE level. There were also no major adverse effects to the treatment. However, there is still a long way to go in exploring specific mechanisms of TCM controlling asthma, especially anti-inflammation and anti-remodeling. The relevant content will be discussed in this review below.

### The Herbal Medicine Formulae in Inhibiting Inflammation and Remodeling

As a mainstay therapeutic approach in TCM, Herbal therapy, or the Traditional Medicine Formulation, is the use of a mixture of herbal ingredients underlying different principles like one emperor, minister, or assistant. The emperor herb fulfills the main therapeutic purpose with the reminders of other herbs in the formulation modulating other effects.

### Bu-Shen-Yi-Qi Fang

Bu-Shen-Yi-Qi fang (BSYQF), a traditional medical formula, has been justified that could suppress chronic airway inflammation according to an *in vitro* study. The whole formulation consists of *Astragalus mongholicus Bunge*, *Rehmannia glutinosa* (Gaertn.) DC., and *Epimedium brevicornu* Maxim. A recent study (Huang M. et al., 2021) has reported that the modified BSYQF can alleviate airways inflammation, AHR, mucus hypersecretion, and collagen deposition via a decrease in the expression of the VIP–VPAC2 signaling pathway in the mouse model. BSYQF has more than 16 chemical constituents including acteoside, catalpol, and icariin. Some of these components have the effect of modulating oxidative stress and reactive oxygen species (ROS) signaling, which are involved in airways inflammation and remodeling in asthma (Chiou et al., 2004; Bi et al., 2008; Kleniewska and Pawliczak, 2017; Sahiner et al., 2018). Aiming to investigate whether BSYQF had an effect on airways remodeling, Cui et al. 2019) sensitized OVA-induced mice model with BSYQF orally. The results indicated that BSYQF treatment reduced airway remodeling including ASM thickening and peribronchial collagen deposition. In terms of oxidative stress, BSYQF treatment decreased the reactive oxygen species (ROS), malondialdehyde (MDA), NO, and restored mitochondrial ultrastructural changes of bronchial epithelia, which indicated the possibility of BSYQF playing an antioxidant role through mitochondrial pathway and eventually inhibited the airway remodeling. Further, after using the RNA-seq analysis to obtain the expressed genes, Cui et al. (2021) also found the expression of several coding genes that had altered in asthmatic models. Based on the gene network by IPA, the study inferred that Adipoq, SPP1, and TNC may contribute to the regulation of BSYQF’s anti-remodeling effect. Therefore, BSYQF is expected to become a hopeful preventive agent against airway inflammation and remodeling for asthma patients.

### Gu-Ben-Fang-Xiao Decoction

Gu-Ben-Fang-Xiao decoction (GBFXD) is a well-known TCM formula derived from a combination of Yupingfeng San (YPFS), a classical formula widely used for treating respiratory diseases, and Erchen decoction. GBFXD has been clinically used in asthma for decades, and the collection of herbal plants can be traced back to Dan Xi Xin Fa, a famous TCM classic. Previous studies (Huang et al., 2016; Lu et al., 2016) have shown that GBFXD can not only downregulate the expression of asthmatic susceptibility genes orosomucoid 1-like protein 3(ORMDL3) and ADAM33 but also inhibit endoplasmic reticulum stress (ERS), which attenuates chronic airway inflammation. A study (Liang et al., 2020) designed to explore if GBFXD offers inhibition against B cell activation in asthma has been conducted. The mice model of asthma was made by the intraperitoneal injection of OVA combined with the intranasal administration of respiratory syncytial virus (RSV). The eventual data showed that GBFXD reduced the level of inflammatory cytokines such as IL-6, IgE, tumor necrosis factor-α(TNF-α), and B cell-activating factor (BAFF), moderated the inflammatory alterations in lung tissues of asthmatic models, and decreased the proportion of BAFF + cell subsets in bronchoalveolar lavage fluid (BALF). In conclusion, the anti-inflammatory functions of GBFXD may be associated with the depression of B cell activation and the release of IgE. Xing et al. (2019). employed the label-free proteomic method to demonstrate the effect of GBFXD treatment in RSV-OVA-induced chronic persistent asthmatic mice and found that GBFXD may control the inflammatory response of asthma via regulating cholesterol transport and complement factor activation. As for structural abnormalities, GBFXD may avoid airway remodeling by possessing the function to repair damaged airway epithelium. According to another study (Liu et al., 2019) that evaluated the effect of GBFXD on asthmatic mice by the iTRAQ labeling technology, it was recorded that several mechanisms, such as improving Th1/Th2 balance, inhibiting alternatively activated macrophages, and suppressing mitochondrial function, are closely associated with the anti-remodeling of GBFXD in asthma.

### Mahuang Decoction

Mahuang Decoction (MHD), a classical multi-herbal prescription from Treatise on Cold Pathogenic Disease (Shang Han Lun in Chinese), has been used for thousands of years to get spasmolysis, diffuse the lung to resolve phlegm, and relieve asthmatic symptoms. The ingredients of MHD (He et al., 2012) consist of *Ephedra sinica Stapf*, *Cinnamomum verum J. Presl*, *Prunus armeniaca* L., and *Glycyrrhiza glabra* L. In a recent study, Huang et al. (2020) determined seven components in MHD *in vivo* by the High-Performance Liquid Chromatography-Mass Spectrometry (HPLC-MS) method and found that amygdalin may play a more vital role in modulating inflammatory cytokine levels of asthmatic mice models. However, the immune mechanism of MHD in asthma is still unknown. Through constructing asthma-related protein–protein interaction (PPI) network based on the DAVID database, Jiao et al. (2018) targeted 20 components in MHD at 32 kinds of proteins in the asthma network and verified it *in vivo*. The results suggested that the key compounds of MHD may regulate asthma via ERK, FceRI, PI3K-Akt, TLRs, and JAK-STAT6 signaling pathways. Additionally, the mitigation of MHD in airway inflammation was proved in another study (He et al., 2018). MHD group significantly restrained the levels of MMP-9, ILs-(2,4,5), and Tissue Inhibitor of Metalloproteinase-1 (TIMP-1) in the rat serum and depressed the protein expression of IL-21, IL-21R, STAT3, and p-STAT3 in lung tissues, which indicate a close joint between the MHD inflammatory efficacy and its regulation of the IL-21/STAT3 signaling pathway in OVA-induced models. Meanwhile, given the monitoring role (Kim et al., 2013) of MMP and TIMP for regulating airway remodeling, the study also demonstrated the possibility of MHD that influences airway repair and remodeling by modulating ECM degradation, thereby inhibiting the thickness of airway walls.

### Others

Apart from the mentioned formulas above, there are 11 decoctions that have been justified for the effect of anti-inflammation or anti-remodeling of asthmatic models *in vivo* or *in vitro*. The efficacy of YPFS is satisfactory in asthma, and YPFS can significantly downregulate the production of IgE. According to the addition and substruction rules of TCM, Jia-Wei-Yu-Ping-Feng-San (JWYPFS) is improved from YPFS to clear the heat and reduce phlegm strongly and applied in OVA challenged mice. JWYPS (Xue et al., 2021) can control the type 2 response mediated by Group 2 innate lymphoid cells (ILC2s), accordingly restoring the expression of inflammatory cytokines IL-5 and IL-13. It is reported that the compound Maqin decoction (CMD) (Xie et al., 2016) and Fangxiao formula (FXT) (Ge et al., 2019) could inhibit the protein production level of Smad-3 in lung tissues, thereby resulting in the alleviated ASM thickness, which suggested the potential clinical effect of them in airway remodeling via TGF-β/Smad signaling pathway. Jin-Gui-Shen-Qi Wan (JGSQW), as a common formula originated from *Synopsis of Prescriptions of the Golden Chamber*, has been clinically utilized to treat various diseases belonging to kidney deficiency such as diabetes (Hu L. et al., 2021), allergic rhinitis (Feng et al., 2021) as well as asthma (Ji et al., 2018). Kao (Kao et al., 2018) surveyed the treatment of JGSQW on chronic asthma by suppressing the *Dermatophagoides pteronyssinus*-induced infiltration of inflammatory cells into airways. Moreover, the study investigated the modulating effect of JGSQW on the NF-κB signaling pathway in epithelial cells and demonstrated that JQSQW could reverse abnormal lung functions by reducing the overexpression of NF-κB-regulated genes (e.g., ICAM-1). Histopathological examinations justified that JGSQW alleviated the airway remodeling in mice models. Another decoction ameliorating Yang deficiency, Yanghe Pingchuan granules (YPG), can restrain the abnormal proliferation of ASMCs (Pan et al., 2018) and control airway remodeling by blocking the PI3K/PKB pathway in asthmatic rats.

Several modified formulas have also been used to improve asthma symptoms clinically for decades in China. For asthma patients in remission, it is shown that the modified Si-Jun-Zi Tang (MSZJT), a multi-herbal decoction, can improve lung function and reduce AHR in asthma. As for the animal study (Jin et al., 2019), MSJZT effectively restrained the major characteristic features of asthma such as AHR, inflammatory cytokine, and especially T effector (Teff) cell levels, which occurred, fully or partly, through inhibiting the Mechanistic Target of Rapamycin 1 (mTORC1) signaling pathway in a chronic murine model of asthma. Xiaochuanping powder (XP) is another TCM formula consisting of medical herbs with confirmed efficacy. Zhou et al. (2018) investigated the effect of XP on asthmatic rats and found that XP could suppress the infiltration as well as the activation of eosinophils, depress the mRNA production of MMP-9 and TIMP-1, and balance the expression between two factors, thereby moderating the inflammation and remodeling of the airways. Based on the TCM theory that “collaterals obstructed by Feng stasis and phlegm” as potential pathogenesis, Yan et al. (2020) verified the effect of Soufeng Yuchuan (SFYC) decoction against airway remodeling in asthmatic rat models. The results suggested that the early application of SFYC decoction in asthma may ameliorate airway remodeling via downregulating the expression of vascular endothelial growth factor (VEGF) as well as TGF-β1. Meanwhile, some patented drugs from TCM have been proved their anti-inflammation effect in asthma. It (Jing et al., 2020) was observed that Qingfei oral liquid (QF) inhibited the Transient Receptor Potential Vanilloid 1 (TRPV1) signal in RSV-infected models. Consequently, QF significantly alleviates asthmatic complications including AHR and mucus hypersecretion. Cheng explored the mechanism of the Yan-Hou-Qing (YHQ) formula in modulating asthmatic inflammation, utilizing the suppression of Th2 response in murine models (Cheng et al., 2019). An *in vitro* study (Wang C. et al., 2019) verified that Pingchunning Decoction plays a vital role in the anti-autophagy mechanism of asthma pathogenesis via activating the PI3K/Akt/mTOR signaling pathway. The whole mechanisms of different formulae are consulted in Table 1.

**TABLE 1:** Associated TCM formulae in inhibiting inflammation and remodeling.

TCM Formulae	Form	Type of study	Animal	Inducer	Mechanism of action	Pathway	References
Bu-Shen-Yi-Qi fang	Decoction	*In vivo*	BALB/c mice	OVA	AHR↓	VIP–VPAC2	Huang et al
					Mucus hypersecretion↓ collagen deposition↓	
		*In vivo*	BALB/c mice	OVA	ASM thickness↓ Peribronchial collagen deposition↓	Antioxidant	Cui et al
					Oxidative stress↓		
		*In vivo*	BALB/c mice	OVA	Abnormal coding gene↓	PI3K/AKT and MAPK	Cui et al
Gu-Ben-Fang-Xiao decoction	Decoction	*In vivo*	BALB/c mice	RSV	Susceptibility gene ↓	ORMDL3	Huang et al
		*In vivo*	BALB/c mice	RSV	ERS↓ Chronic airway inflammation↓	PERK and IRE1α	Lu et al
		*In vivo*	BALB/c mice	RSV-OVA	Inflammatory cytokines↓ Asthmatic alterations↓	B cell	Liang et al
		*In vivo*	SPF mice	RSV-OVA	Regulate cholesterol transport and complement factor activation	RAF/MEK/ERK	Xing et al
		*In vivo*	BALB/c mice	OVA	Fit the pattern of an alternative M2 activation state, AHR↓	M2 macrophage	Liu et al
Mahuang Decoction	Decoction	*In vivo*	SD rats	OVA	Inflammatory cytokines↓		Huang et al
		*In vivo*	BALB/c mice	OVA	Sensitization time↓ Abdominal breathing time↓	TLR9	Jiao et al
		*In vivo*	SD rats	OVA	Related protein expression↓	IL-21/STAT3	He et al
Jia-Wei-Yu-Ping-Feng-San (JWYPFS)	Powder	*In vivo*	C57BL/6 mice	OVA	Inflammatory cytokines↓	ILC2s	Xue et al
Maqin decoction (CMD)	Decoction	*In vivo*	SD rats	OVA	Smad3↓	TGF-β/Smad	Xie et al
Fangxiao formula (FXT)	Decoction	*In vivo*	SD rats	OVA	Smad3↓	TGF-β/Smad	Ge et al
Jin-Gui-Shen-Qi Wan (JGSQW)	Pilula	*In vivo*	BALB/c mice	Der p	Airway inflammatory infiltration↓ Reverse abnormal lung function	NF-κB	Kao et al
Yanghe Pingchuan granules (YPG)	Decoction	*In vivo*	SD rats	OVA	ASMCs↓	PI3K/PKB	Pan et al
Modified Si-Jun-Zi Tang (MSZJT)	Decoction	*In vivo*	BALB/c mice	OVA	AHR ↓ Inflammatory cytokine levels↓	mTORC1	Jin et al
Xiaochuanping powder (XP)	Powder	*In vivo*	SD rats	OVA	Infiltration↓ Eosinophils↓ Recover the balance between the expression of MMP-9 and TIMP-1	MMP/TIMP	Zhou et al
Soufeng Yuchuan (SFYC) decoction	Decoction	*In vivo*	SD rats	OVA	General condition↑ Lung damages↓	VEGF/TGF-β1	Yan et al
Qingfei oral liquid (QF)	Decoction	*In vivo*	BALB/c mice	RSV	AHR↓ Mucus hypersecretion↓	TRPV1	Jing et al
Yan-Hou-Qing (YHQ) formula	Decoction	*In vivo*	BALB/c mice	OVA	Asthmatic symptoms↓	Th2	Cheng et al
Pingchunning Decoction	Decoction	*In vitro*	SD rats	OVA	Pulmonary pathology↓ Autophagy↓	PI3K/Akt/mTOR	Wang et al

↑:increase; ↓:decrease; OVA: ovalbumin; AHR: airway hyperresponsiveness; ASM: airway smooth muscle; VIP- VPAC2: vasoactive intestinal polypeptide-type 2 VIP, receptor; Der p:Dermatophagoides pteronyssinus; PI3K/AKT: phosphatidylinositol 3-kinase and protein kinase B; MAPK: mitogen-activated protein kinase; RSV: respiratory syncytial virus; ORMDL3: orosomucoid 1-like protein 3; ERS: endoplasmic reticulum stress; PERK: protein kinase RNAlike ER, kinase; IRE1α: inositol-requiring enzyme 1α; TLR9: Toll-like receptor 9; IL-21: interleukin-21; STAT3: Signal Transducers and Activators of Transcription 3; TGF-β: transforming growth factor-beta; NF-κB: nuclear factor kappa-light-chain-enhancer of activated B-cells; mTORC1: Mechanistic Target of Rapamycin 1; MMP: matrix metalloprotein; TIMP: Tissue Inhibitor of Metalloproteinase; VEGF: vascular-endothelial-growth-factor; TRPV1:Transient-Receptor-Potential-Vanilloid-1.

### The Traditional Herbs in Inhibiting Airway Inflammation and Remodeling

As the most basic therapeutical unit in Chinese medical formulations, the herbs from TCM have been given great expectations for preventing or even treating a couple of diseases. But to find out the directed association between classical TCM therapies and pharmacodynamic evidence, an increasing number of molecular studies have focused on the individual herbs or herbal extracts. Specifically, it is investigated that several herbs are beneficial to alleviate asthmatic airway inflammation and remodeling in murine models.

Actaea cimicifuga L. is a common herbal remedy documented in editions of the pharmacopoeia of China. The dried roots of cimicifuga, known as *Cimicifuga heracleifolia* Kom., have been vastly utilized as the source of Chinese herbal medicine Sheng Ma in terms of its antipyretic, anti-inflammatory, and antioxidative effects (Niu et al., 2019). To evaluate the efficacy of Cimicifuga Rhizoma extract (CRE) on asthma, the mice (Lim et al., 2021; Pang et al., 2021) were administrated CRE orally at 30 and 100 mg/kg. The mice’s inflammatory alterations, including the expression of pro-inflammatory cytokines and production of mucus, have been successfully attenuated at a dose of 100 mg/kg. In addition, CRE may upregulate the level of antioxidant proteins (e.g., HO-1, Nrf2) to protect against OVA-induced asthmatic inflammation and oxidate stress. Besides, Hu Z. et al. (2021) classified 110 chemical compounds from CRE and investigated their immunomodulatory activity evaluation through *in vivo* and *in vitro* studies as well. Based on the data of the study profile, it is proved that CRE could prevent neutrophil infiltration in the lung tissues against asthmatic chronic inflammation. In spite of that little research reported on the role of anti-airway remodeling induced by cimicifuga, the present findings still suggest that the treatment of cimicifuga might be a novel alternative therapy for asthmatic airway symptoms.

As a classic tonic for the deficiency of lung, the Cordyceps, including *Cordyceps sinensis* as well as its common substitute *Cordyceps militarisis*, has been widely used for medicine in the treatment of respiratory diseases. From previous research (Tan et al., 2020), one of the key compounds from cordyceps, cordycepin, has been prized against various inflammatory lung injuries owing to its multiple bioactivities such as accelerating the immunity and suppressing the cytokine storms. Moreover, the *in vivo* study (Chiou and Lin, 2012) indicated that the extract of cordyceps could remarkedly modulate the airway inflammation in a murine model through downregulating the activity of NF-κB. In terms of asthma, it (Wang et al., 2016) is reported that the administration of *Cordyceps sinensis* alleviated asthma symptoms, lung function, and especially accelerated the health-related quality of life of patients with moderate-to-severe persistent asthma in a clinical randomized controlled trial. Additionally, by restraining the activation of the TGF-β1/Smad pathway, Cordyceps polysaccharide^0^ (Zheng et al., 2020) extracted from *Cordyceps militarisis* could attenuate goblet cell hyperplasia and inflammatory cell infiltration so as to alleviate the OVA-induced AHR in a murine model. The present research cannot provide a direct proof that Cordyceps may alleviate airway remodeling in asthma, but Cordyceps (Yang et al., 2018) has been verified its potential efficacy in moderating the thickness of airway walls in rats with COPD, which indicated that the treatment of Cordyceps may become a useful approach for asthma patients.

With promising pharmacological findings meriting further experiments and clinical studies, an increasing number of Chinese herbal medicine have shown a unique bioactive effect in managing asthmatic small airway symptoms or alterations. Both ethanolic and water extract (Luo et al., 2019) from *Salvia miltiorrhiza Bunge* (S. miltiorrhiza) could significantly inhibit inflammatory cell infiltration, goblet cell hyperplasia, and restore the level of Th1/Th2 cytokines. Interestingly, the study indicated that S. miltiorrhiza water extract is more valid in alleviating airway remodeling and responsiveness. Wang Q. et al. (2021) investigated that the total flavonoids from Qu zhi qiao, known as the fruit of *Citrus aurantium* L., may regulate the Smad2/3 and MAPK signaling to improve the negative effect induced by airway structural alteration and remodeling. Simultaneously, the extracts from *Perilla frutescens* (L.) Britton (Zisu) (Yang H. et al., 2020), *Asparagus cochinchinensis* (Lour.) Merr (Tianmendong) (Sung et al., 2017), and *Leonurus japonicus* Houtt (Yimucao) (Wieczfinska et al., 2020) have also verified their possibilities as a suppressor in airway inflammation and remodeling in asthmatic murine models. The more detailed mechanisms of herbs’ efficacy are consulted in Table 2.

**TABLE 2:** Major anti-airway inflammation and remodeling mechanisms of the traditional herbs.

TCM herbs	Origin	Method of study	Animal or cell	Mechanism of action	References
Shengma	*Actaea cimicifuga L*	HPLC–DAD		Antipyretic, inflammation↓ oxidative effects↓	Niu et al
		*In vitro*/*In vivo*	RAW264.7 cells/SD rats	iNOS↓	Pang et al
				Inflammatory activities↓	
		*In vivo*	BALB/c mice	Nrf2/HO-1/NQO1↑ NF-κB↓	Lim et al
		*In vitro*/*In vivo*	BEAS-2B cells/SD rats	Neutrophil’s infiltration↓	Hu et al
Dong Chongxiacao	*Cordyceps sinensis* and *Cordyceps militarisis*	*In vitro*/*In vivo*	A549 cell/SD rats	Accelerate the immunity cytokine storms↓	Tan et al
		*In vivo*	BALB/c mice	Modulate the airway inflammation NF-κB↓	Chiou et al
		RCT		Alleviate asthma symptoms lung function↑ health-related quality of life↑	Wang et al
		*In vivo*	BALB/c mice	Goblet cell hyperplasia↓ Inflammatory cells infiltration↓	Zheng et al
		*In vivo*	Wistar rats	The thickness of airway walls↓	Yang et al
Danshen	*Salvia miltiorrhiza Bunge*	*In vivo*	BALB/c mice	Inflammatory cell infiltration↓ Goblet cell hyperplasia↓ Restore the level of Th1/Th2 cytokines	Luo et al
Qu zhi qiao	*Citrus aurantium L*	*In vivo*	BALB/c mice	Smad2/3↓ MAPK↓	Wang et al
Zisu	*Perilla frutescens* (L.) Britton	*In vivo*	BALB/c mice	Modulate airway inflammation Syk↑	Yang et al
Tianmendong	*Asparagus cochinchinensis (Lour.) Merr*	*In vivo*	BALB/c mice	Recover histopathological structure inflammatory mediators↓ IgE↓ IL-4↓ IL-13↓ COX-2↓	Sung et al
Yimucao	*Leonurus japonicus Houtt*	*In vivo*	WI-38 and HFL1 cells	Influence airway remodeling process MMP-9↓ TGF-β↓	Wieczfinska et al

↑:increase; ↓:decrease; HPLC–DAD: high-performance liquid chromatography coupled with diode array detection; iNOS: inducible nitric oxide synthase; Nrf2/HO-1/NQO1: Nuclear Transcription Factor 2/Heme Oxygenase 1/Recombinant NADH, dehydrogenase, Quinone 1; NF-κB: nuclear factor kappa-light-chain-enhancer of activated B-cells; RCT: randomized controlled trial; MAPK: mitogen-activated protein kinase; Syk: Spleen TyrosineKinase; IL-4:Interleukin-4; IL-13:Interleukin-13; COX-2:cyclooxygenase-2; MMP-9:matrix-metalloprotein-9; TGF-β:transforming-growth-factor-beta; WI-38:Wistar-Institute-38; HFL1:human-fetal-lung-fibroblast.

### The Function of Natural Compounds From TCM Against Airway Inflammation and Remodeling

The investigation of the mechanisms of the multi-target effect of TCM is extremely vital to developing anti-asthma new drugs. In recent years, multiple active ingredients from Chinese herbs have been recognized as the potential basis of TCM treatment with a diversity of pharmacological effects. To better explore and clarify the effectiveness of TCM in asthmatic inflammation and airway remodeling, researchers have tested the efficacy of herbal compounds *in vivo* or *in vitro*; thereby, the active ingredients are usually regarded as shooting stars in the study of new drugs. These ingredients are normally divided into different categories, including saponins, alkaloids, ketone, flavonoids, quinone, and others, which will be discussed separately below.

### The Saponins

Glycyrrhizic acid (GA) is known as one of the most crucial signature bioactive constituents isolated from the traditional Chinese herb *Glycyrrhiza uralensis* Fisch. ex DC., where its anti-inflammatory activity has also been proved in a past study (Yang et al., 2017). GA (Yao and Fu, 2021) can significantly alleviate airway inflammation, inflammatory cell infiltration, and remodeling *in vitro* and markedly restore the thickness of ASM and airway walls *in vivo*, which is at least partly associated with the modulation of the TGF-β1/Smad signaling pathway. It is worth noting that the study was the first to investigate that GA may suppress chronic asthma symptoms. Similarly, the cycloastragenol (CAG), hydrolyzed from the roots of *Astragalus mongholicus* Bunge, has been widely utilized to exert an anti-inflammatory effect in clinical study. The asthmatic mice models^0^ were established by the immune of OVA and the study (Zhu et al., 2021) demonstrated that the CAG ameliorated AHR as well as the secretion of inflammatory cytokines *in vivo*. Furthermore, it is shown that both the autophagic flux and the expression level of autophagy-associated proteins reduced after the treatment of CAG, which suggested the relationship between CAG and the inhibition of autophagy in lung cells. The root of *Panax ginseng* is a classic herb in TCM for the treatment of asthma with lung-qi deficiency syndrome. Among the biologically active compounds extracted from *Panax ginseng* C.A.Mey., Ginsenosides are believed as the main effective ones consisting of Rb1, Rb3, Rg1, Rc, Rh2, Rg2, and Rg3 (Kim, 2018). Ginsenoside Rg1 (Chen et al., 2015) can efficiently relieve AHR and airway inflammation through regulating Th2 activity in a mice model. Moreover, it (Guan et al., 2020) is reported that the administration of Rg1 inhibits inflammatory responses via modulating the TGF-β1/Smad3 signaling pathway, thereby improving lung function and against cigarette smoke-induced airway remodeling. In terms of reducing anti-oxidative stress, the present study (Huang W. C. et al., 2021) demonstrated that the application of Ginsenoside Rg3 has significantly positive potential as a regulator in asthmatic mice. It has notably restrained eosinophil infiltration and mucus hypersecretion in mice lung tissues and exerted its anti-inflammation effect by suppressing the expression of Th2 cytokine and eotaxin expressions.

### The Alkaloid

As a symbolic bioactive compound, the alkaloid widely exists in Chinese herbal medicine. Some of which have been verified for their clinical efficacy after animal experiments. Cordycepin (Cor) is a commonly used ingredient for the treatment of asthma and originated from *Cordyceps militaris*, a traditional herbal mushroom, in Chinese medicine practice. In order to identify the target of Cor regulating airway remodeling, Fei et al. (2017) have compared the level of inflammatory cytokines in OVA-induced rats BALF between the Cor group, glucocorticoids group, and combination group. The result indicated that the treatment of Cor may up-regulate the transcription of A_2A_ARmRNA as well as inhibit the generation of TGF-β1. Further, the data also revealed that the Cor exerts its anti-remodeling effect primarily by suppressing the expression of the p38MAPK signaling pathways. It is reported that Sinomenine (Sin), a bioactive alkaloid isolated from the root of *Sinomenium acutum* (Thunb.) Rehder & E.H.Wilson, has an effective restrained effect on airway remodeling and inflammation in mice with asthma. Sin (He et al., 2021) not only hinders the expression of MMP7, MMP9, and vimentin *in vivo* but inhibits the EMT progress by modulating the IL-4 levels in the serum, which alleviates the airway inflammation in the mice lung. Meanwhile, the study has shown that the Sin decreases the synthesis of TGF-β1 and Smad3 and attenuates subepithelial collagen deposition, which has a positive effect on relieving airway remodeling in asthmatic mice. In another study (Işık et al., 2018), the dose of 100 mg/kg Sin treatment has been investigated that provided effective improvement on all of the histopathological outcomes about airway remodeling compared to placebo (*p* < 0.05). The levels of cytokines in BALF, serum, or immunohistochemical scores have also markedly reduced in the 100 mg/kg Sin-induced group, which suggested that Sin efficacy may associate with its modulating effect on Th-2-derived cytokines and apoptosis of airway epithelial cells. Ligustrazine (LTZ) is extracted from a traditional medicinal herb *Ligusticum striatum* DC., which has been widely used in clinical practice to clear the heat and remove the stasis in blood. It (Liu et al., 2018) is reported that LTZ may play a positive role in alleviating asthmatic inflammatory symptoms by upregulating the secretion of suppressed factors IL-10 but downregulating promoting factors IL-17 in murine models of neutrophilic asthma. It has been suggested that imbalanced oxidative stress and antioxidant defense involve in the airway remodeling in asthma.

Additionally, several bioactive alkaloids, such as Piperlongumine (Lu et al., 2019), Matrine (Yu et al., 2019), and Oxysophocarpine (Li G. et al., 2020), have been adequately justified for their anti-inflammatory activities in murine models via inhibiting NF-κB or MAPK signaling pathway. Apart from these, the alkaloid from TCM also displayed double-treated advantages both in airway inflammation and remodeling. Tetrandrine (Tet) isolated from the roots of *Stephania tetrandra* S. Moore can suppress alveolar inflammatory infiltration, airway remodeling, and the expression of CysLT1 and CysLTR1 in OVA-sensitive rats and inhibit the cell viability of ASM cells *in vitro* (Lin et al., 2019). Therefore, Tet may alleviate airway remodeling by affecting TGF-β1/Nrf-2/HO-1 signaling cascades and become a novel drug candidate for patients with chronic asthma. In clinical practice, *Tetradium ruticarpum* (A.Juss.) T.G.Hartley, including its extractive Evodiamine, is a classic medicine in China for treating the deficiency of Yang, especially in the kidney. Considering the synergy between kidney and lung and bioactivities of Evodiamine, Wang S. et al. (2021) established the AI(OH)_3_- and OVA-induced rat models to evaluate its potential protective effect against asthma. The study reported that the administration of Evodiamine can alert the level of IgE, reduce the expression of the HMGB1 gene, and the infiltration of inflammatory cells in rat lungs. Moreover, compared with the untreated group, the treatment of Evodiamine reduces mucus secretion, the thickness of both the airway wall and the smooth muscle layer, and collagen deposition in asthmatic rats, which profited from the knockdown of HMGB1/NF-κB/TLR-4. As a diterpenoid alkaloid extracted from *Aconitum carmichaeli Debeaux* plants, Bulleyaconitine A (BLA) is investigated that it (Liu L. et al., 2020) can suppress the excretion of IgE, IL-17A, and Th2 cytokines, but increase the excretion of Th1 cytokines to recover the Th1/Th2 balance in murine BALF and serum. These results eventually showed that BLA may have a positive role against AHR, lung inflammation, and airway remodeling. However, whether BLA has an anti-pain or bronchodilation activity should be evaluated in further study.

### The Ketone

Icariin (ICA) is one of the most major active ingredients of the Chinese traditional herb *Epimedium brevicornum Maxim* and has been given special consideration in respect of its various bioactivities including enhancing humoral immunity, relieving asthmatic cough, anti-oxidant, and anti-allergic (Sze et al., 2010; Qiao et al., 2017). A couple of studies have been carried out to estimate the effects and mechanisms of ICA in airway inflammation and remodeling of asthma. ICA (Hu et al., 2019) can attenuate ASM proliferation and key factors of the MAPK/Erk pathway *in vitro* and downregulate the increase of IL-13 as well as endothelin-1 in serum and BALF of murine models exposed to OVA *in vivo*, which indicates a bright future that ICA as replacement therapy for asthmatic patients. On the other hand, Li Z. et al. (2020) investigated the underlying mechanism of ICA alleviating EMT in asthma *in vivo* and *in vitro*. The TGF-β induced the EMT progress including migration, and the up-regulation of N-cadherin and α-SMA in 16HBE cells has also been inhibited by ICA. The administration of ICA could restrain the phosphorylation of Smad-2, Smad-3, JAK, Erk, and p38 at the molecular level, thereby inhibiting the activation of the Smad and MAPK signaling pathway. Further, it (Tian et al., 2020) is demonstrated that icariside II could moderate airway inflammation and remodeling induced by eosinophils *in vitro* and attenuate the cell proliferation and migration in ASMCs via NF-κB and STAT3 signaling. These data reveal the exact reasons for the use of epimedium originated herbal medicine in TCM to gain ideal efficacy in treating asthma. Curcumin, one diketone compound, is well known for its anti-inflammatory potential in clinical. Kumari et al. (2019) treated the LPS- and OVA-exposed mice with curcumin through an intranasal way to evaluate its efficacy against airway structural alterations. The corresponding study indicates that intranasal curcumin may inhibit detrimental airway remodeling in lungs by means of regulating key signaling pathways and significantly suppressing inflammatory mediators as well as related proteins such as MMP-9 or TLR-4. In recent research (Zhang Y. et al., 2021), baicalin isolated from the roots of *Scutellaria baicalensis Georgi* has shown its protective effect on cigarette smoke-induced airway inflammation in COPD rats’ models, which is partially through modulating the HDAC2/NF-κB/PAI-1 signaling. Another animal study (Yuan et al., 2018) also suggested that the treatment of curcumin may have a positive effect on airway inflammation and remodeling in the COPD model, which is closely associated with the suppression of the BEAS-2B cell proliferation and the activation of NF-κB and COX-2, whereas these findings figured out a possibility that curcumin and baicalin are potential agents in the therapy of asthma.

In addition to modulating inflammatory signaling pathways, the ketone compounds still alleviate airway inflammation and remodeling in other ways. *Astragalus mongholicus Bunge* has been widely utilized in traditional Chinese medicine to benefit the flow of Qi and improve the deficiency of Qi, and formononetin (FMT) is the main isoflavone obtained from it. Yi et al. (2020) justified that the treatment of FMT remarkedly subsided goblet cell hyperplasia and collagen deposition and simultaneously recovered the imbalance of oxidation and antioxidation as displayed by the inhibited reactive oxygen species (ROS) but stimulated the activity of superoxide dismutase (SOD). Similarly, to FMT, luteolin is a typical flavonoid compound that has been proved to its anti-inflammatory and immune regulating effects. Furthermore, luteolin (Wang W. et al., 2021) restrained the level of autophagy of lung tissues in OVA-induced mice models with the partial mechanism including activating the PI3K/Akt/mTOR signaling pathway and downregulating the beclin-1–PI3KC3 protein complex. In conclusion, the underlying mechanisms of the ketone compounds provide a solid basis for their clinical application in the treatment of asthmatic patients.

### Others

The multiple natural compounds from TCM have gotten in the intervention of asthmatic airway inflammation and remodeling individually or jointly. It is approved that andrographolide (AG) plays a vital role in the anti-inflammation treatment of various pulmonary diseases such as cough and asthma. However, whether the related capacity of AG exerts by regulating the immunologic function is unknown. Thus, Yu et al. (2021) established the asthmatic mice model induced by OVA with AG treatment, and the conclusion is that AG could significantly attenuate the airway remodeling and the neutrophil infiltration of the lung tissue and block the activation of T17 cells. Interestingly, at the molecular level, another *in vivo* study (Xia et al., 2019) demonstrated that AG antagonizes CS-induced EMT progress as well as pulmonary dysfunction by restraining the IL-6/STAT3 pathway. Curcumol is a classic herbal monomer isolated from *Curcuma phaeocaulis Valeton* for its regulatory effects on pulmonary fibrosis and oxidate stress (Li L. et al., 2020). The present study (Jia et al., 2021) revealed that curcumol could obstruct the abnormal activation of the Wnt/β-catenin pathway in turn to moderate the chronic lung inflammation and airway remodeling, which also explained the relief of symptoms in murine models.

Rheum palmatum, L. is used as an herbaceous plant for thousands of years in China, and it has been determined that anthraquinone derivatives are its main pharmacodynamic compounds such as emodin or chrysophano. Most corresponding studies about emodin that have been published paid more attention on the anti-inflammatory effect on the asthmatic airway. An example of this is the study carried out by Hua et al. (2019) in which emodin decreased the expression of inflammatory cytokines (e.g., IL-5, IL-17) in BALF and serum of mice, which is partially related to the downregulation of the Notch pathway. However, aiming to explore the underlying mechanism of emodin in ASMCs, the allergic mice models were established by OVA exposure. The findings (Liu Y. et al., 2020) eventually indicated that the intraperitoneal injection of emodin at 20 mg/kg moderated the thickness of ASM in lung tissues and AHR as well. Additionally, the emodin alleviated ASMC proliferation in a dose-dependent manner through blocking the PI3K/Akt pathway *in vivo* and *in vitro*. Similarly, another active constituent from Rheum, chrysophanol, has shown its potential effect on asthma-associated lung inflammation and airway remodeling *in vivo* and *in vitro*. Chrysophanol (Song et al., 2019) can improve the abnormal autophagy in the mice lung induced by OVA and inhibit the proliferation from the cell level via the NF-kB signaling pathway. Both the anti-autophagy and antiproliferation effects of chrysophanol contribute to ameliorating asthmatic symptoms.

It has been revealed that cryptotanshinone (CTS) extracted from *Salvia miltiorrhiza* Bunge, a well-known Chinese medicine in terms of restoring the stasis of blood in the lung, could exert multiple bioactivities such as protecting LPS-induced lung injury in murine models (Tang et al., 2014). As for asthma, Li J. et al. (2020) found that the administration of CTS successfully alleviates the asthmatic airway inflammation in the way of recovering the secretion balance between Th1 and Th2 cytokines. Wang X. et al. (2019) investigated that CTS not only reduced the accumulation of inflammatory cells and the level of OVA-specific IgE in BALFs but also fulfilled a similar effect as a tumor necrosis factor-like weak inducer of apoptosis (TWEAK) inhibitor, which helps to relieve airway remodeling. Shikonin, a naphthoquinone, is isolated from the roots of *Arnebia euchroma* (Royle ex Benth.) I.M.Johnst., and its prohibitive effect (Wang et al., 2017) on the proliferation and migration of ASMCs was similar to the pyrrolidine dithiocarbamate (PDTC), an inhibitor of the NF-κB pathway, which may uncover the potential mechanism of shikonin treating airway inflammation and remodeling in asthma.

At present, aside from the discussed compounds above, there still occur various natural inhibitors from TCM with the potential efficacy against airway inflammation and remodeling in asthma. It is demonstrated in most cases that the active ingredients can markedly suppress the production of the inflammatory cells or cytokines and decreased the thickness of airway walls or smooth muscle, which at least partly makes a contribution to the alleviation of asthmatic inflammation and airway remodeling (e.g., resveratrol, osthole, and imperatorin) (Jiang et al., 2019; Yang Q. et al., 2020; Xian et al., 2020). Additionally, through OVA-induced murine models, Zeng et al. (2019) have justified the protective effect of polydatin (PD) for pulmonary injury in asthma by accelerating Nrf2-mediated antioxidation to recover the EMT progress of lung epithelial cells, in which PD treatment moderated the asthmatic reactive oxygen species (ROS) and airway remodeling. Chen et al. (2021) revealed that Schisandrin B (SB) could effectively block the activation of NLRP3 inflammasome and undermine pyroptosis from the molecular level, thereby improving the symptoms of asthmatic mice. The mechanism of components mentioned above is consulted in Table 3.

**TABLE 3:** The active components from TCM against airway inflammation and remodeling.

Species	Components	Origin	Mechanism of action	Pathway	References
Saponins	Glycyrrhizic acid	*Glycyrrhiza uralensis Fisch. ex DC.*	Exhibit its anti-inflammatory properties; the thickness of ASM↓ Airway walls↓	TNF/MMP	Yang et al
					Yao et al
	Cycloastragenol	*Astragalus mongholicus Bunge*	AHR↓ inflammatory cytokines↓	Autophagy	Zhu et al
			Autophagic flux↓		
			Autophagy-associated proteins↓		
	Ginsenoside Rg1	*Panax ginseng C.A.Mey*	Relieve airway inflammation AHR↓ Th2 activity↓	Th2; TGF-β1/Smad3	Chen et al
			Inflammatory responses↓		Guan et al
	Ginsenoside Rg3	*Panax ginseng C.A.Mey*	Th2 cytokine↓	Th2	Huang et al
			Eotaxin expressions↓		
Alkaloid	Cordycepin	*Cordyceps militaris*	A_2A_ARmRNA↑	TGF-β1/MAPK	Fei et al
	Sinomenine	*Sinomenium acutum (Thunb.) Rehder & E.H.Wilson*	Decrease the synthesis of related cytokines	MMP; TGF-β1/Smad3	He et al
			EMT↓ Th2-derived cytokines↓		Işık et al
			Apoptosis of airway epithelial cells↓		
	Ligustrazine	*Ligusticum striatum DC.*	IL-10↑ IL-17↓	IL	Liu et al
	Piperlongumine	*Piper longum L*	Inhibit TNF-α-induced inflammatory cytokine expression	NF-κB	Lu et al
	Matrine	*Sophora flavescens Aiton*	Neutrophil apoptosis↓		Yu et al.0
	Oxysophocarpine	*Sophora flavescens Aiton*	Cell apoptosis↓	miR-155	Li et al.0
			Inflammatory infiltration ↓		
	Tetrandrine	*Stephania tetrandra* S. Moore	ASM cells↓	TGF-β1/Nrf-2/HO-1	Lin et al
			CysLT1↓		
			CysLTR1↓		
	Evodiamine	*Tetradium ruticarpum (A.Juss.) T.G.Hartley*	Mucus secretion↓ the thickness of airway wall↓	HMGB1/NF-κB/TLR-4	Wang et al
			the smooth muscle layers↓		
			Collagen deposition↓		
	Bulleyaconitine A	*Aconitum carmichaeli Debeaux*	Recover the Th1/Th2 balance		Liu et al
Ketone	Icariin	*Epimedium brevicornum Maxim*	Exert anti-oxidant ability by active endogenous scavenging enzymes; PGD2↓ ASM↓ EMT↓ attenuate the cell proliferation and migration in ASMCs	CRTH2; MAPK/Erk; Smad; NF-κB/STAT3	Sze et al
					Qiao et al
					Hu et al
					Li et al
					Tian et al
	Curcumin	*Curcuma longa L*	Regulate inflammatory mediators	MMP-9/TLR-4	Kumari et al
	Baicalin	*Scutellaria baicalensis Georgi*	Protect lung tissues	HDAC2/NF-κB/PAI-1	Zhang et al
	Curcumin	*Scutellaria baicalensis Georgi*	Cell’s proliferation↓	NF-κB	Yuan et al
	Formononetin	*Astragalus mongholicus Bunge*	Goblet cell hyperplasia↓		Yi et al
			Collagen deposition↓		
			ROS↓ SOD↑		
	Luteolin	*Lonicera japonica Thunb*	Autophagy↓	PI3K/Akt/mTOR	Wang et al
Terpene	Andrographolide	*Andrographis paniculata (Burm. F.) Nees*	Attenuate neutrophil infiltration of lung tissue T17 cells↓	IL-6/STAT3	Yu et al
			EMT progress↓		Xia et al
	Curcumol	*Curcuma phaeocaulis Valeton*	Obstruct the abnormal signaling Pulmonary fibrosis↓ oxidate stress↓	Wnt/β-catenin	Li et al
					Jia et al
Anthraquinone	Emodin	*Rheum palmatum L.*	Inflammatory cytokines↓	Notch; PI3K/Akt	Liu et al
			The thickness of ASM↓		
	Chrysophanol	*Rheum palmatum L.*	Abnormal autophagy↓ Proliferation↓	NF-kB	Song et al
Phenanthraquinone	Cryptotanshinone	*Salvia miltiorrhiza* Bunge	Protect lung injury; recover the secretion balance between Th1 and Th2 cytokines; IgE↓	TWEAK	Tang et al
					Li et al
					Wang et al
Naphthoquinone	Shikonin	*Arnebia euchroma (Royle ex Benth.) I.M.Johnst*	The proliferation and migration of ASMCs↓	NF-kB	Wang et al
Polyphenol	Resveratrol	*Reynoutria japonica Houtt*	Inflammatory cytokines↓	HMGB1/TLR4/NF-κB	Jiang et al
	Polydatin	*Reynoutria japonica Houtt*	Recover the EMT progress	Nrf2	Zeng et al
Coumarin	Osthole	*Cnidium monnieri (L.) Cusson*	Restore the release of inflammatory cytokines	IL-33/ST2	Yang et al
	Imperatorin	*Kitagawia praeruptora (Dunn) Pimenov*	Inflammatory cytokines↓	Nrf2/HO-1	Xian et al
Lignin	Schisandrin B	*Schisandra chinensis (Turcz.) Baill*	Pyroptosis↓	NLRP3	Chen et al

↑:increase; ↓:decrease; TNF: tumor necrosis factor; MMP: matrix metallopeptidase; ASM: airway smooth muscle; AHR: airway hyperresponsiveness; TGF-β1: transforming growth factor-beta; MAPK: mitogen-activated protein kinase; EMT: Epithelial–Mesenchymal Transition; IL: interleukin; TNF-α: Tumor Necrosis Factor-α; NF-κB: nuclear factor kappa-light-chain-enhancer of activated B-cells; CysLTR1: Cysteinyl Leukotriene Receptor 1; Nrf-2/HO-1: Nuclear Transcription Factor 2/Heme Oxygenase 1; HMGB1: High Mobility Group Box 1; TLR-4: Toll-Like Receptor 4; CRTH2: known as prostaglandin D2 receptor 2; STAT3: Signal Transducer And Activator Of Transcription 3; HDAC2: Histone Deacetylase 2; ROS: reactive oxygen species; SOD: superoxide dismutase; PI3K: phosphatidylinositol-4, 5-bisphosphate 3-kinase; TWEAK: tumor necrosis factor-like weak inducer of apoptosis; NLRP3: NLR, Family Pyrin Domain Containing 3.

Thus, the cores of these *in vivo* and *in vitro* findings are the same, which highlight the potential mechanism of natural compounds inhibiting airway inflammation as well as remodeling, and preferring novel insights for the treatment of asthma.

## Conclusion and Outlook

Presently, bronchial inflammation and airway remodeling as the common pathologic basis shared by asthma have severely threatened the prognosis and life quality of chronic patients. Specifically, small airway dysfunction including inflammatory cell infiltration and airway structural alteration is still the major difficulty in the treatment of asthmatic symptoms and depression of lung function. Current anti-asthma therapies consisting of ICS with LABA and updated monoclonal antibodies can only manage symptoms but do not alter sensitization or stabilize the milieu interne. However, as one of the major alternative and complementary medicines worldwide, TCM has multiple unique advantages against asthmatic inflammation and airway remodeling in clinic based on the *in vivo* and *in vitro* evidence.

**FIGURE 2:**
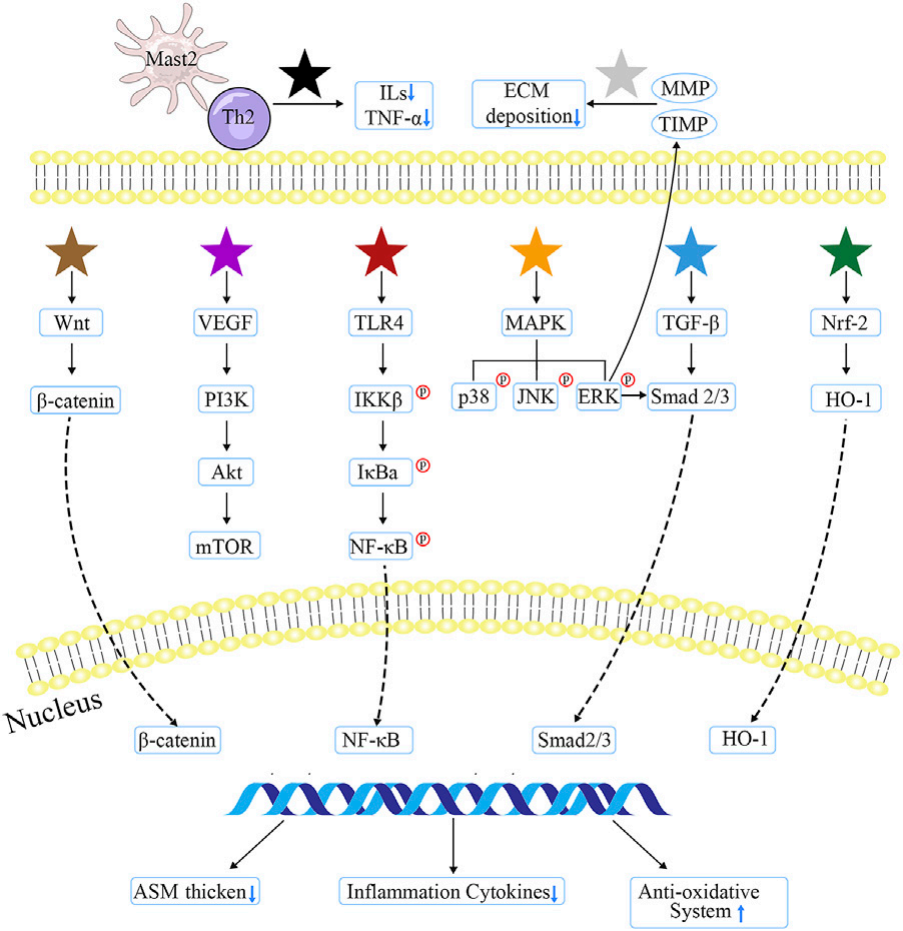
The major targets and mechanisms of TCM involved in anti-airway inflammation and remodeling. Black star indicates Gu-Ben-Fang-Xiao decoction, Jia-Wei-Yu-Ping-Feng-San, ginsenoside Rg1/3, ligustrazine, bulleyaconitine A, andrographolide, and osthole; the gray one represents Xiaochuanping powder, glycyrrhizic acid, and curcumin; brown star signifies curcumol; purple star means Yanghe Pingchuan granules, Soufeng Yuchuan decoction, Pingchunning Decoction, luteolin, and emodin; red star refers to Mahuang Decoction, Jin-Gui-Shen-Qi Wan, piperlongumine, evodiamine, baicalin, curcumin, chrysophanol, shikonin, and resveratrol; orange star denotes Bu-Shen-Yi-Qi fangm cordycepin, and icariin; blue star implies Mahuang Decoction, Fangxiao formula, and sinomenine; green star suggests tetrandrine, polydatin, and imperatorin.

After thoroughly reviewing these evidence, the major targets and pathways that formulae, herbs and natural compounds from TCM suppressing airway alterations can be classified into several types: Mast 2/Th2 cell type (e.g., BSYQF, ligustrazine, etc.) can alter the M2/Th2 activation state, thereby downregulating the cytokines’ level; MMP/TIMP type (e.g., XP, glycyrrhizic acid, etc.) may reduce EMT deposition by regulating MMP or TIMP; Wnt/β-catenin type (e.g., curcumol, etc.) would obstruct abnormal signaling pathway in order to alleviate pulmonary symptoms; VEGF/PI3K type (e.g., YPG, emodin, etc.) depresses inflammatory cytokines through blocking VEGF or PI3K pathways; TLR/NF-κB type (e.g., JGSQW, evodiamine, etc.) may involve the most anti-inflammatory natural compounds that restore mucus secretion; MAPK type (e.g., BSYQF, icariin, etc.) could exert an anti-remodeling ability, which was also associated with EMT; TGF-β1 type (e.g., CMD, sinomenine, etc.) inhibits the thickness of ASM and airway walls; and Nrf2/HO-1 type (e.g., tetrandrine, imperatorin, etc.) mainly improves the oxidative effects. The detailed mechanisms of TCM are consulted in Figure 2.

Above all, the mechanisms of how TCM alleviates bronchial inflammation and airway remodeling include but are not limited to blocking the activation of inflammatory cells, regulating various immune cytokines’ level, reducing ECM progress in lung tissues, and AHR among big or small airways through different signaling pathways. From the molecular level, autophagy, as the core progress of cell self-renewal, has received increasing attention to participate in the regulation of several major human disorders (Klionsky et al., 2021) including diabetes, cancer, and pulmonary disorders. Accumulating evidence suggest that autophagy activators from TCM (e.g., formula and bioactive extracts) are potential therapeutical candidates and promising strategies for asthmatic inflammation and airway remodeling treatment. Related research (Lambrecht and Hammad, 2012) has justified that the stress responses of airway epithelial cells are a major culprit in asthma and proved the benefit in the modulation of cytokines. Given the critical target cytokines that TCM altered mostly involved in the epithelium network, systematic TCM treatment may restore the innate epithelial response therapy to prevent airway remodeling and asthma exacerbations.

Although we have found that various decoctions, natural compounds, and extracts from TCM displayed anti-inflammation and anti-airway remodeling effects in different murine models, we are still far away from translating these TCM approaches into broad clinical applications for patients suffering from vital asthma. At present, the establishment of asthmatic animal models was normally induced by allergic factors such as OVA but not available for non-allergic factors, which also generate a wide range of airway inflammation and tissue structural changes (Fang et al., 2020). The property of asthma types should be taken into account, or which may weaken the efficacy of TCM in clinical application. Moreover, the above findings were mostly derived from *in vivo* or *in vitro* experiments, and the current clinical studies cannot provide valid support for the conclusion. Thus, not only large-scale randomized controlled tests or retrospective cohort studies but the deeper pharmacological and theoretical research on TCM should be carried out to verify its safety, efficacy, and exact mechanisms in the future.

In conclusion, though there are still some issues that need to find the solution, it is still demonstrated from enumerated research that TCM has wide potential application and promising effect in managing small airway dysfunction in asthma. We believe that with further investigation focusing on Chinese traditional herbs and formulas, TCM will definitely offer more Eastern wisdom to cure human disorders.
